# A Comprehensive Study of Progressive Cytogenetic Alterations in Clear Cell Renal Cell Carcinoma and a New Model for ccRCC Tumorigenesis and Progression

**DOI:** 10.1155/2010/428325

**Published:** 2010-07-05

**Authors:** Zhongfa Zhang, Bill Wondergem, Karl Dykema

**Affiliations:** ^1^Laboratory of Cancer Genetics, Van Andel Research Institute, Grand Rapids, MI 49503, USA; ^2^Center for Systems and Computational Biology, The Wistar Institute, Philadelphia, PA 19104, USA; ^3^Computational Biology, Van Andel Research Institute, Grand Rapids, MI 49503, USA

## Abstract

We present a comprehensive study of cytogenetic alterations that occur during the progression of clear cell renal cell carcinoma (ccRCC). We used high-density high-throughput Affymetrix 100 K SNP arrays to obtain the whole genome SNP copy number information from 71 pretreatment tissue samples with RCC tumors; of those, 42 samples were of human ccRCC subtype. We analyzed patterns of cytogenetic loss and gain from different RCC subtypes and in particular, different stages and grades of ccRCC tumors, using a novel algorithm that we have designed. Based on patterns of cytogenetic alterations in chromosomal regions with frequent losses and gains, we inferred the involvement of candidate genes from these regions in ccRCC tumorigenesis and development. We then proposed a new model of ccRCC tumorigenesis and progression. Our study serves as a comprehensive overview of cytogenetic alterations in a collection of 572 ccRCC tumors from diversified studies and should facilitate the search for specific genes associated with the disease.

## 1. Introduction

Cytogenetic changes underlie most genetic diseases, including cancer. Not only are different patterns of cytogenetic alterations associated with different types of tumors, tumors of the same type at different stages of development also exhibit different cytogenetic patterns. It is commonly accepted that tumors at early stages of development have fewer cytogenetic alterations than tumors at more advanced stages. Early mutations in a few key genes are believed to drive the initial steps of tumorigenesis. Key mutational events are loss-of-function mutations in tumor suppressor genes (TSGs) and/or gain-of-function mutations in oncogenes. The resulting changes in gene function are thought to trigger the process of tumorigenesis and set the stage for the accumulation of more genetic abnormalities as the tumor progresses. Here we have treated gene methylation as a special form of mutation, for ease of description.

Mutations are rare. In the two-hit theory of cancer formation [1], loss of TSG function occurs in two stages: mutation of one of the two parental gene copies occurs as the first “hit”, followed by a second hit when a large-or-small-scale chromosomal deletion or a structural alteration inactivates the remaining TSG allele. Thus, TSGs are often contained in regions having copy number loss. On the other hand, an oncogene often gains its functionality via an increase in gene copy number or, in some cases, activating mutations of the gene. Therefore, an oncogene is more likely to be contained in regions where copy numbers of DNA sequences are amplified. Analysis of cytogenetic losses and gains in tumor cells can thus identify potential tumor suppressors and oncogenes. 

Advances in technology now allow for cytogenetic analysis in unprecedented detail. Detailed cytogenetic analysis of a given tumor type at different stages of progression will be necessary for a full understanding of tumor development. Cytogenetic profiling of different tumor subtypes can also shed light on the understanding and diagnosis of cancer by revealing genetic alterations specific for each subtype. In this study, we undertook a comprehensive survey of cytogenetic losses and gains occurring in renal cell carcinoma (RCC) by using the high throughput high density Affymetrix 100 K SNP chips. 

RCC is a heterogeneous disease consisting of multiple subtypes. The most common RCC subtype is clear cell (ccRCC), accounting for about 70% of all RCC tumors. Other subtypes include papillary (PA, ~10%), chromophobe (CH, ~5%), oncocytoma (ON, <1%), and collecting duct carcinomas (very rare) [2, 3]. Despite large-scale genomic screening, the only gene that is commonly mutated is the *von Hippel-Lindau (VHL) *gene [4]. Mutations, methylation, and/or LOH of the *VHL* gene are frequently observed in ccRCC tumors (~90%). Cytogenetic profiling of various RCC subtypes has been studied indirectly through genome-wide gene expression profiling [2, 5, 6]. Traditional methods such as comparative genomic hybridization (CGH) [7–12], quantitative PCR [13], fluorescent in situ hybridization (FISH), and cytogenetic banding [14, 15] have also been applied. Only recently has the use of high-throughput high density DNA microarray chips become available for detailed genome-wide cytogenetic alteration study. These microarrays offer higher resolution than traditional methods. For example, we recently used such high density microarrays to profile genome-wide cytogenetic alterations in ccRCC and papillary RCC [16] and Beroukhim et al. combined both 500 K SNP data and gene expression data to identify the cytogenetic alterations in VHL and sporadic tumors [17]. However, little is known about the specific sequence by which genetic alterations take place during ccRCC progression. The most relevant study to our current one is from Jiang et al., who relied on comparative genomic hybridization data to construct an evolutionary tree model for RCC [18]. To our knowledge, there have been no systematic studies characterizing progressive cytogenetic alterations in ccRCC development using high-throughput high-density 100K SNP microarray data. 

Here we report a systematic study of cytogenetic alteration profiles in ccRCC tumors at different stages of development and in association with tumor grades. Our study provides a detailed map of the sequence of cytogenetic alterations that occur during the progression of ccRCC. Based on these results, we propose a new model of ccRCC tumor genesis and progression. Our results suggest the involvement of cytogenetic alterations seen in ccRCC, and will aid in the identification of genes relevant to ccRCC.

## 2. Material and Methods

### 2.1. Tissue Collection

71 pretreatment tissue samples were collected from multiple cancer centers, including Spectrum Health Hospital in Grand Rapids, MI (31 cases), and the Cooperative Human Tissue Network (CHTN, 38 cases) over the past few years. There were 42 cases of ccRCC, 6 cases of chromophobe, 8 cases of oncocytoma, and 15 cases of papillary RCC. Clinicopathological information for the 42 ccRCC patients was summarized in Table 1. All tumors were primary and sporadic in that the patients did not have a known familial history of their tumor types. The tissues were pulverized under liquid nitrogen and genomic DNA was prepared by a standard protocol using proteinase K/SDS lysis followed by phenol/chloroform extraction and ethanol precipitation. The clinicopathological diagnosis of tumors followed the standard TNM diagnosis of RCC. Tumor staging and grading as well as other pathological factors (e.g., vein or vascular invasions, necrosis or sarcomatoid factors, etc.) were obtained from review of the pathology staining reports and evaluation of the case notes by individual clinicians. In particular, the difference between T1a and T1b tumors is largely in the tumor size. Readers can refer to the American Cancer Society for more information on tumor staging criteria (http://www.cancer.org/). VHL mutations were obtained through sequencing of all three exons of the gene. 

In addition to our current study, we also selected 5 large-scale cytogenetic studies in the literature for a combined study. Out of the 5 studies, 3 had tumor stage information, 2 did not. There were 572 ccRCC samples in total with different techniques used to detect cytogenetic alterations. The clinicopathological information was summarized in Table 1. The copy number analysis based on tumor stages was summarized in Table 2. 

### 2.2. DNA Preparation

We used Affymetrix 100K SNP chips to obtain genomic information. Each 100K SNP chip contains two subchips using either *Xba*I or *Hin*dIII to digest the genome materials. Digestion of the DNA, PCR, and scanning followed the instructions from Affymetrix (http://www.affymetrix.com/). All data samples passed the quality controls both before application to the chip and after scanning the hybridized chip.

### 2.3. SNP Data Analysis

Raw SNP copy numbers were calculated based on CNAG software version 2 [21]. We used a total of 56 normal samples to obtain the normal copy number 2 signal. Among them, 48 were downloaded from Affymetrix and 8 were obtained from our own scans on normal kidney tissues that we have collected. The raw copy numbers were replaced by running *t*-test statistics (with a window size of 31 SNPs for the moving windows). The *t*-test makes use of the copy number's local constant property, which assumes that the losses or gains of SNP copy numbers tend to happen in segments. Neighboring SNPs tend to have the same copy numbers. Thus, by borrowing the information from its neighbors, this copy number calculation will increase its power of uncovering the underlying DNA alterations and decrease noise. This operation is equivalent to smoothing the calculated raw copy numbers. The correction is necessary because DNA samples of tumor tissues prepared for SNP runs were generally not a homogenous collection of tumor cells, but rather a mixed collection of tumor and other cells (e.g., stroma). Even the tumor cells may have different cytogenetic patterns with differed combinations of numbers of signal for the target over signals for the control (such as 3 : 2, 2 : 3, etc.). For example, the low-grade and early-stage tumors tend to have a lower proportion of tumor cells than the high-grade and later-stage tumors. We call this phenomenon partial gain or partial loss, as opposed to complete gain or loss in that all cells used for microarray scan are tumor cells. As a result, without any corrections, the final raw copy numbers of the tumor cells would be a weighted mixture distribution of all the components of cells prepared for the scan, usually pulled toward the normal copy number of 2. A solid cutoff to claim a gain or loss will be improper if one takes this into consideration.

### 2.4. Statistical Methods

The smoothed copy numbers were then summarized based on cytoband using the regional expression bias (*reb*) package in Bioconductor [22], adapted to the SNP data. Briefly, the algorithm grouped the probes by the associated SNP locations; for each region, a general test (such as binomial or *t*-test) was applied to determine if the raw copy numbers in the region were collectively higher or lower than that of normal. The test statistics were then output for each tumor sample and for each cytogenetic region. Disease-specific survival was used and was defined to be the time from first operation date to either death or last known follow-up date. For each cytogenetic region with cross sample interquartile range of the summarized cytogenetic alteration scores (the scores were the output from algorithm *reb* for each tumor and for each cytogenetic region) greater than 2.5, a number of survival models were built to associate the patients' survival with the summarized cytogenetic alteration scores. We set the number of models to be 100 for each cytoband. Each model was built on a randomly selected but fixed number of subset of ccRCC patients. The score test was used to calculate the prognostic significance of *P*-values for the association. The transformed *P*-values (−log10(*p*)) were averaged over that from the 100 models as the final significance of association for the cytoband, as well as the regression coefficients. A studentized test was used to test the difference of summarized cytogenetic alterations between two groups of samples.

### 2.5. Display of Copy Number Gains or Losses for a Group of Samples through Boxplot

**Figure 1:**
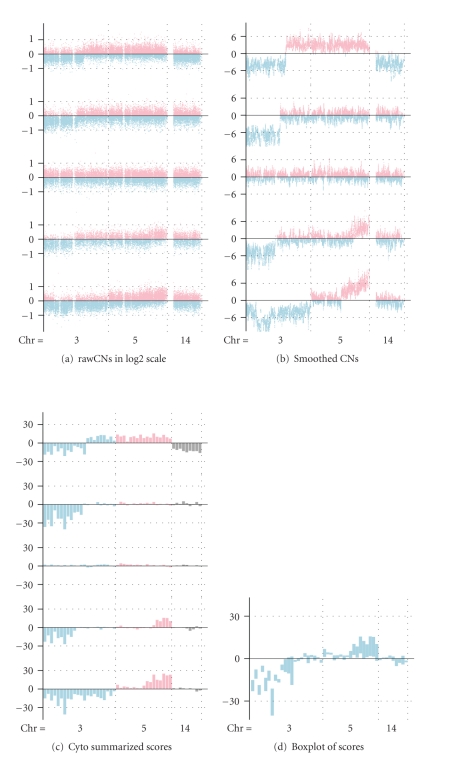
Illustration of algorithm for obtaining and displaying the cytoband summarized SNP copy number scores for a group of samples. (a) Display of raw SNP copy number alterations for individual tumor samples. Each horizontal line in the figure represents data from one tumor sample (total of five tumor samples displayed). Raw SNP copy numbers are displayed in log 2 scale and plotted on the *Y*-axis. Negative copy numbers (indicating SNP losses) are depicted in blue and positive copy numbers (indicating SNP gain) are depicted in pink. The *X*-axis represents the physical ordering of individual SNPs along different chromosomes. (b) Display of smoothed SNP copy number alterations for individual tumor samples. Each horizontal line represents data from one tumor sample (total of five tumor samples displayed). (b) the smoothed copy number by moving *t*-test with window size 31, (c) the cytoband summarized SNP copy number scores using the adapted regional expression bias algorithm, and (d) boxplot of the summarized SNP copy number scores.

To display the collective copy number gains or losses for a group of samples, we first calculated for each sample and for each cytoband, the summarization scores of copy numbers of SNPs within the cytoband through *reb* algorithm, which was described previously on the smoothed copy numbers. A positive value represents a gain, while a negative value represents a loss for the cytoband. The absolute values represent the degrees of losses or gains. The data from each cytoband over all samples in the group were then summarized and a box was produced to represent the summarization. The boxes were placed side by side ordered by their physical positions on the chromosome, from p-arm to q-arm, from chromosome 1 to 22. We omitted analysis for chromosome X. The upper and the lower bounds of the boxes are the third and first quartiles of the data sequences. If the box is well below zero, it indicates that the majority of the samples in the group have losses in their copy numbers in the cytoband (at least 75%). If the box is well above zero, it is interpreted as majority of the samples in the group have gains in their copy numbers (at least 75% again). Thus the boxes contain both the frequency and the intensity of the gain or loss events in the group. It is a natural combination of information for both. The vertical length of the box reflects the range of sample CNA (cytogenetic alteration numbers) values: the longer the box, the more variable the range of sample values. Thus, short box lengths reflect a tighter clustering of SNP copy number values than do longer box lengths. The midpoint of each box represents the median CNA value for all samples. An illustration figure was displayed in Figure 1. 

### 2.6. Determination of Losses or Gains of Chromosome Arms

To determine loss or gain of a specific chromosome arm for a specific sample to produce Table 2 for the current study, we used a cutoff value of 10 on the cytoband summarized copy number data that were output from running the *reb* algorithm. A value larger than 10 was marked a gain for the whole cytoband for the sample, while a value less than −10 was marked a loss for the cytoband. The proportions of losses or gains for each cytoband across all tumors in the study group were calculated. The proportion of loss (gain) of an arm was determined to be the highest loss (gain) proportion among all cytobands within the arm. If the proportion of loss was higher than that of gain, the arm was an overall loss and vice versa (undecided cases when the loss proportion equals to the gain proportion are unlikely to happen for the selected arms). The numbers and proportions of losses or gains for selected chromosome arms calculated this way were then summarized in Table 2.

## 3. Results

### 3.1. RCC Tumor Subtypes have Distinct Cytogenetic Alteration Profiles

**Figure 2:**
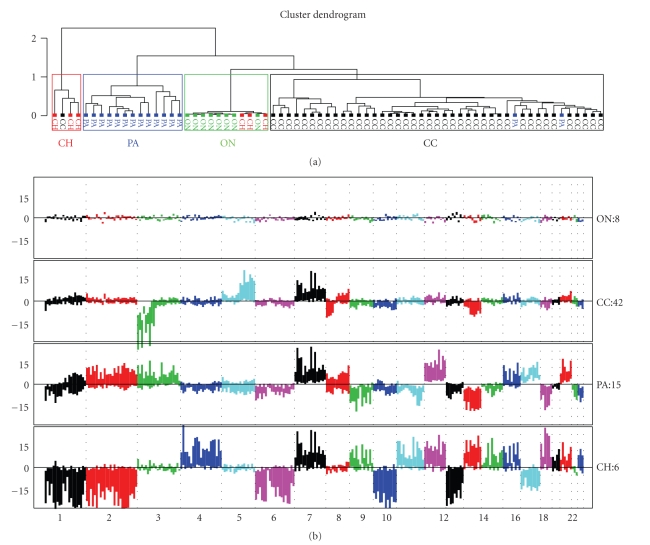
(a) Unsupervised clustering of RCC samples based on cytoband summarized SNP data. CH: chromophobe RCC, PA: papillary RCC; ON: oncocytoma, CC: clear cell. With few exceptions, tumors of a given subclass clustered together. Thus, each of the four RCC subtypes has a distinct pattern of cytogenetic alterations. (b) Somatic cytogenetic alteration profiles of RCC subtypes. RCC subtypes are ordered by complexity of molecular cytogenetic alterations. Each bar in the plot is a box with upper and lower bound of the box representing the third (75%, upper bound) and first quantiles (25%, lower bound) over the summarized SNP copy numbers by cgma method. The boxes are ordered from p-arm to q-arm followed by another chromosome. Only the somatic chromosomes are shown here. A positive value stands for gain, while a negative value stands for loss. Each chromosome is assigned a single color different from its neighbor chromosomes.

First, we compared the cytogenetic profiles of the four RCC subtypes: clear cell, papillary, chromophobe, and oncocytoma (Figures 2(a) and 2(b)). Unsupervised clustering of tumor samples based on their cytogenetic data revealed four clusters in the plot, roughly corresponding to the four RCC subtypes. Tumors of a given subtype generally clustered together. For example, all tumors of clear cell type clustered together, with the exception of one case which clustered with chromophobe samples. Our results indicate that each RCC subtype displays a distinct cytogenetic alteration profile (Figure 2(a)). This cytogenetic profiling correctly predicted tumor subtypes with an overall accuracy of 92% (65/71). Although we cannot be certain, we speculate that tumors misclassified by this analysis had mixed features or were misdiagnosed.

### 3.2. −3p, +5q, and −8p Are Unique Events for ccRCC

Comparing cytogenetic profiles of four RCC tumor subtypes, we found that −3p, +5q and −8p are unique to ccRCC tumors (Figure 2(b)). Tumor subtypes were listed in increasing order of cytogenetic complexity, from having the least alterations (oncocytoma) to the most ones (chromophobe). Apart from oncocytoma, which is a benign tumor and does not possess any obvious cytogenetic changes, +7, +12, and −16 were seen to occur in all other RCC subtypes, but to varying degrees. This suggests there may be common cytogenetic alterations affecting shared signaling pathways in these RCC subtypes. Interestingly enough, we observed that there were no regions having copy number losses which were common to all three malignant RCC tumors. −14q was seen in ccRCC and papillary RCC only, while +14q was seen in chromophobe RCC. −3p was seen in ccRCC tumors only, while +3p was seen in papillary RCC tumors and 3p was unchanged in chromophobe RCC. Therefore, it is likely that each RCC subtypes has a distinct tumor initiation (such as gene mutations) patterns, especially for TSGs if they are involved in the development of RCCs. As the *VHL* gene is located on 3p25-26, this indicates that deregulation of *VHL* pathway is involved for ccRCC development only, but not for the other tumor subtypes. Thus, different RCC subtypes have unique cytogenetic alterations as well as common alterations. As ccRCC accounts for the majority of RCC tumors, we will focus our analysis on this subtype only in the rest of paper. 

### 3.3. −3p, +5q, +7, and −14q Are Associated with Early Tumor Stages of ccRCC

**Figure 3:**
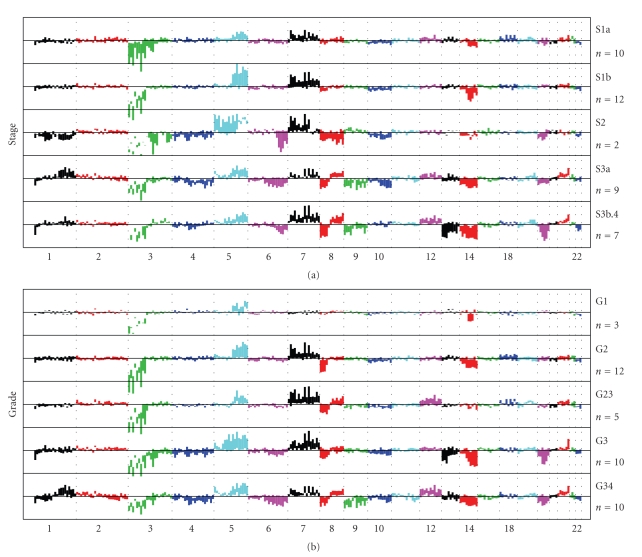
(a) Somatic cytogenetic alteration summarization based on SNP data, grouped by patient tumor stages in increasing order. Box plots were arranged as in Figure 2. The earliest stage S1a shows the fewest cytogenetic alterations, while the highest stage S3b4 shows the largest number of cytogenetic changes, indicating the cytogenetic progression of ccRCC tumors. −3p, +5q, +7, −8p, and −14 are among the earliest events during ccRCC tumorigenesis, while −1p, +1q, −4p, −6q, −9p, +12, −13, −18, and +20q are events occurring at later stages only. (b) Somatic cytogenetic alterations associated with tumor grade. Box plots are arranged as in Figure 2.

Tumor stage is an important factor in determining tumor progression. We grouped ccRCC tumors and analyzed their cytogenetic alteration profiling according to tumor stages (Figure 3(a)). As was expected, we saw that cytogenetic alteration events increased as tumor stage increased. Tumors at the earliest stage, S1a, had the least cytogenetic alterations with −3p, +5q, +7, and −14 only, while tumors at the latest stages, S3b + S4 (the only case of stage 4 tumor was merged with S3b) had the most cytogenetic alterations, notably −1p, +1q, −3p, +5q, −6q, +7, −8p, +8q, −9, +12, −13, −14, −18, +20, and −22. This agrees with the prevailing model of tumorigenesis and progression: as tumor progresses, initial simple genetic events (sequence mutations, segmental losses or gains) trigger more and more cytogenetic alterations through altering the tumor cell microenvironment, causing the tumor genome to become less stable. Loss in chromosome 17 has been previously reported to be associated with later stage events of RCC [23]; however, in our samples, we did not find obvious loss or gain of whole and/or part of chromosome 17. 

### 3.4. −3p and +5q Are the Most Frequent Events for ccRCC Tumors and Are Weakly Related

Our data suggest that −3p and +5q are universal events in all stages of ccRCC tumors (Figure 3(a)) and in all grades of tumors (Figure 3(b)). In other word, they appeared to be unrelated to tumor stage and grade. In later stages, losses in 3p were largely unchanged in frequencies (around 70%–80%, Table 2), but gains in 5q occurred with varied frequencies and degrees (Table 2 and Figure 3(a)). The two events appeared to be weakly inversely related to each other (Pearson's correlation test with *correlation coefficient = −0.25*, *P* =.12). As unbalanced translocations between 3p and 5q were frequently observed [24], this result implies the possibility of 5q gain without 3p loss or vice versa. Indeed, Podolski and colleagues studied 5q gains intensely, found that there was a high frequency in an unbalanced translocation between 3p and 5q, leading to the 3p loss of one of its parental copies and 5q gains. 5q gain could also occur in independent ways to 3p loss, such as to have unbalanced translocations with other chromosomes [25]. The most frequent losses on 3p were 3p26, 3p24, 3p14 and 3p21-22 for the early-stage tumors (Figure 3(a) and Supplementary Figure S1A in supplementary material available online at doi:10.1155/2010/428325, detailed plot of 3p is not shown). +5q occurred most frequently for stage 1a tumors at 5q31-5qter. It reached its peak at intermediate stages (S1b and S2), then started to decrease at later stages of S3 and S4. This observation gives partial support of the previously reported result that 5q gain was associated with good prognosis of ccRCC patients [24, 25]. We will discuss this in more detail in the following section.

### 3.5. 5q Gain Is Likely Involved in the Transition from Stage 1a to Stage 1b Tumors and May Play a Critical Role in ccRCC Development

The profiles of cytogenetic alterations between stage 1a and stage 1b tumors were remarkably similar (See Figure 3(a) and Supplementary Figures S1A, S1B). A formal studentized *t*-test was used to test the difference of cytogenetic alterations between the two groups of tumors having stages 1a and 1b. A test statistic below −2 or above 2 was used to declare the differences to be significant. The profile of test *P*-values was plotted in Supplementary Figure S1C. The identified cytogenetic alterations declared to be significant between tumor groups S1a and S1b were 3p (p25–p22), 5q (q34-q35), 4q23, 11q24 and 17q22. Apart from the single cytoband on chromosomes 4, 11, and 17, the most significant differences were recognized as more gains in 5q and more losses in 3p in stage 1b tumors than in stage 1a tumors. Unlike gains in chromosome 7, +5q is seen to be unique to ccRCC tumors (Figure 2(b)); it occurs in a large proportion of ccRCC tumors (43%, Table 3). To figure out in more details, we then used a 3 cm and 7 cm as criteria for grouping the tumors into one of the small-sized (<3 cm), medium-sized and large-sized (>7 cm) groups. The cytogenetic alteration profile of the 9 small-sized tumors was compared with those of medium- or large-sized tumors (Supplementary Figures 2D–2F. The profiles for the small and medium sized tumors were remarkably similar, with the only striking difference occurred in 5q, where there were no obvious gains in the small sized tumors and where there were a significant more 5q gains in the medium sized tumors. Therefore, a critical role can be proposed for genes in this region during ccRCC formation, whose gene disruptions promote the tumor growth in size and differentiate the kidney tumor cells into well-defined clear cell tumors. Cancer-relevant genes on 5q include the LOX gene, which has been shown to play an essential role in hypoxia-induced metastasis [30], the later is triggered by the disrupted VHL gene in the tumor cells. Other genes in the area include *PDGFRB, *which plays an important role in tumor neovascularization [43]*, TGFBI [16], PTTG1, DOCK2 *and *DUSP1* (the latter three genes were speculated based on our internal studies), which were likely to be involved in ccRCC progression and development. However, no mutation genes were found in this region so far.

### 3.6. 14q Likely Contains Important TSG Genes Unrelated to 3p Loss

As we see from our data, −14q occurred in the earliest stage and lowest grade tumors too (along with −3p, Figures 3(a) and 2(b)); it displayed a clear increasing pattern in occurrence frequencies along tumor stages, uncorrelated with that of −3p occurrences (Table 3). This suggests that −14q is likely to have occurred in an independent way to 3p loss; it also indicates that its occurrences are also influenced by the tumor cell environment: as later and more advanced tumors tend to have more occurrences of −14q. Tumor initiation and promotion roles in ccRCC tumor development can be proposed for genes in this region. Disruptions of one or more than one key TSG genes in this region were likely involved during the early ccRCC tumorigenesis. At the same time, some functionally important genes may also be activated by other events of tumors; those genes may play a significant role in promoting tumor invasiveness, as was shown in Supplementary Figure S2I and S2J. 14q contains functionally diversified genes, critical to a few important and well-studied pathways, such as HIF1*α* (14q21-q24, [44]) and EGLN3(PHD3, 14q13.1, [45]), both of which were shown to be disrupted in ccRCC tumors, due to the disrupted VHL gene functions. The heavy chains of human antibodies (or immunoglobulins) are also located in the far end of 14q, with often elevated activities in ccRCC tumors. On the other hand, other known involved tumor suppressor genes in this region were AKT1 (14q32.32, [46]) and SERPINA5 (14q32, [31]). AKT1 gene was shown to play a pivotal role in the AKT/PI3K signaling pathway. In summary, our data suggest that genes in 14q play important and diversified roles in both ccRCC formation and its development. Again, much remains unknown about the roles the genes in this region play during ccRCC formation and development. More study of genes in the region is needed to better understand the ccRCC tumorigenesis and progression.

### 3.7. Gain of Whole or Part of Chromosome 7 Is an Early Stage Event of ccRCC, Independent of Tumor stage

Gain of chromosome 7 was seen to appear in all stages (Figure 3(a)), indicating that one or more genes on 7 may play an important role in both tumor development and tumor growth. Gain of chromosome 7 also occurred in tumors of all grades except grade 1 (Figure 3(b)). The most active cytoband was identified to be 7q21–7q31 (data not shown). These regions contain a number of functionally important genes, including *MET* (also being called *HGFR, RCCP2, c-Met*) in 7q31, *HGF* in 7q21, *EPO *(Human erythropoietin gene) in 7q22, *VGF* (nerve growth factor inducible gene) in 7q31 and PDGFA (platelet-derived growth factor alpha) in 7p22 or IGFBP1 (7p13-p12). Amplification of or activating mutations in these genes may play a role in the development of ccRCC. Unlike +5q, +7 was not unique to ccRCC, but that it occurs in all malignant RCCs; a general role for genes in this region can be suggested during RCC development, such as proliferation and vascular invasion (Supplementary Figure S2J), leading to poor patients' survivals (Supplementary Figure S2A).

### 3.8. −1pter, +1q, −4 −6q, +8q, −9, +12, −13, −18, and +20 Are Later Stage Events in ccRCC

These events did not appear in the early stage S1a or S1b tumors, but occurred progressively as tumors advance in stage. For example, −13 and −18 occurred only when tumor stages exceeded 2, and they peaked at the latest stage (S3b4, Figure 3(a)). These events also occurred in high-grade tumors only (see Figure 3(b)). This pattern suggests that these cytogenetic alterations are generally the consequences of tumor development due to increased genomic material instability, rather than the cause of it. However, these genetic alterations may still play critical roles in tumor progression, metastasis, and/or proliferation. In a recent study [14], the authors found that cytogenetic alterations of −4/4p and −9/9p, among other alterations, were significantly associated with patients' survivals. We will discuss more about these regions in the coming sections.

### 3.9. Association between Cytogenetic Alterations in ccRCC and Tumor Grade

Next, we grouped and analyzed cytogenetic alterations in ccRCC by tumor grade (Figure 3(b)). The patterns were very similar to that grouped by tumor stage. −3p, +5q, and −14q were seen to appear in all grades of ccRCC tumors. On the other hand, −1p36, −4, −6, +12, −13, −18 and +20 were seen in high-grade tumors only. Of the three tumors with grade 1, we did not see obvious +7 or −8p. −8p appeared to occur after −3p, −14q, and +5q. Due to the small size of grade 1 tumors (3 cases), the clear display of −8p in grade 2 tumors (12 cases), and relative high frequency of occurrence in clear cell tumors (24%, see Table 3), we decide that −8p is a moderate early event in the process of ccRCC tumorigenesis. We noted subtle differences between the patterns of cytogenetic alterations associated with tumor grade compared to alteration patterns with tumor stage. +7 was seen in stage 1a tumors, yet it was not seen in grade 1 tumors. This discrepancy may reflect differences in the clinical features of tumor grade and stage.

### 3.10. Association of Cytogenetic Alterations in ccRCC with Patient Survivals and Other Clinical or Pathological Factors

Next, we examined cytogenetic alterations in ccRCC tumors in association with patient survivals. The averaged significant *P*-values for each cytoband were plotted in Supplementary Figure S2A. Cytobands from chromosomes 1, 2, 4, 6, 7, 8, and 13 were identified to be associated with patients' survivals. Specific cytogenetic bands associated with patient survival were summarized in Supplementary Table 1. Among these regions, −4p/4 and −13 were seen to be most significantly associated with poor survivals. This was confirmed in previous study [14]. +1q was seen to be correlated with quite a few clinicopathological factors, which include sarcomatoid element, stage, vein, and vascular invasions. It is no surprise that +1q is associated with patients' survivals (Supplementary Figure S2). Other regions were also identified to have significant prognostic significance, which were summarized in Supplementary Table 1. Due to our limited number of available cases who have follow-up data, the results displayed here are likely to be conservative. 

Associations between cytogenetic alterations in ccRCC and other clinicopathological features, including VHL mutation status, gender, tumor size, sarcomatoid elements, gross tumor necrosis, renal vein and vascular invasions were examined too; the significances of tests were summarized in Supplementary Table 2 and displayed in Supplementary Figure S2. Of the different profiles associated with different clinical or pathological factors, the profiles for tumor grade (Grade 1-2 versus Grade 3-4, Figure S2D), for tumor stage (Stage 1-2 versus Stage 3-4, Figure S2E) and for tumor size (size < versus ≥4 cm,Figure S2F) are comparably similar, while the profiles for vein invasion (Yes versus No, Figure S2I) and for vascular invasion (Yes versus No, Figure S2J) are remarkably similar.These similarities reflect the closeness of being clinicopathological factors. We note that, interestingly, VHL mutation status is not associated with any specific cytogenetic changes and that +1q is associated with sarcomatoid differentiation, tumor vein and vascular invasions as well as poor patient survivals, indicating that some genes in this gene rich area play critical roles in tumor progression or metastasis. The cytogenetic profiles between male and female patients were remarkably similar too (Figure S2C), except that male patients were more likely to have amplified chromosome 7 and 1q than their female counterparts, and that female patients were more likely to exhibit 8p loss. Further studies were needed to verify if these differences were gender related.

## 4. Discussion

We have studied the whole genome cytogenetic alterations in RCC tumors using high-density high-throughput Affymetrix SNP arrays. We confirmed that different subtypes of RCC had distinct cytogenetic profiles, reflecting the heterogeneity of RCC. We also showed that specific cytogenetic alterations in ccRCC are associated with specific clinicopathological features. Finally, by examining cytogenetic profiles from ccRCC tumors at different stages of progression, we will be able to construct a detailed map of the sequential cytogenetic changes that occur during ccRCC progression below. 

**Figure 4:**
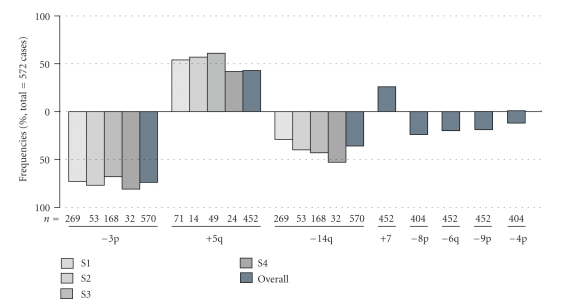
Relative frequencies of cytogenetic alterations in 572 ccRCC tumors from multiple sources.

Based on results in Table 2 and Figure 4 of the combined studies, the most frequent alterations for ccRCC were identified as −3p(74%), +5q(43%), −14q(36%), +7(26%), and −8p(24%). In the four studies where tumor stage information was available, −3p seemed to display no patterns of decrease or increase in frequency as tumor stage increases, while −14q occurrences show an increasing pattern in frequency along tumor stages. In all studies, −14q occurred in at least 20% of stage 1 tumors (average: 29%), then increased in occurrence frequencies as tumor stage increases. This indicates that −14q is among the earliest events in the tumorigenesis process. In our study, −8p occurred in ccRCC tumors only and occurred in almost all grade and stage tumors. This comparison indicates that −8p may have occurred in early but not earlier than −3p and −14q did.

First of all, based on our experience and mouse model study as well as published papers [47], we postulate that there exist more than one independent pathway for ccRCC tumors to form. The majority of ccRCC tumors involve loss of function of the VHL gene, a key regulator of the hypoxia-response pathway. Loss of function of VHL leads to unregulated activity of HIF, a hypoxia-inducible transcription factor. Overactivity of HIF, in turn, leads to uncontrolled activation of the hypoxia-response pathway. The VHL gene has been reported as mutated or methylated in over 70% of ccRCC tumors [48]. However, studies in our laboratory (data not shown) and that in others indicate that more than 90% of ccRCC tumors exhibit deregulation of the VHL pathway. This suggests an independent and parallel event(s) to *VHL* gene mutation for deactivation of the VHL functional pathway. Deactivation of VHL pathway alone is not sufficient to cause the ccRCC phenotype [49, 50].

**Figure 5:**
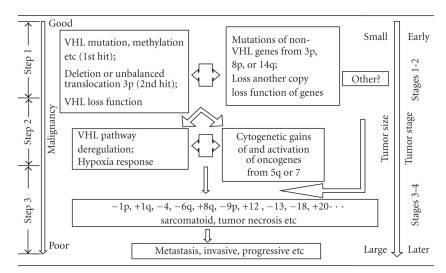
Illustration of a proposed model of ccRCC tumor formation and progression.

Combining all the information, we have collected, we propose a new model of ccRCC tumorigenesis and progression. This model is illustrated in Figure 5. For the majority of ccRCC tumors where VHL is involved, the first step starts when a key event (mutations, methylation, etc.) occurs in VHL, leading to deactivation of the VHL pathway and unregulated hypoxia response. Thereafter, the tumor cells are constantly under altered microenvironment, paving the way toward tumorigenesis. If later on, some key non-VHL TSG genes from 3p, 8p, and/or 14q undergo mutations or gene alterations, leading to the losses of the functions of the corresponding genes, the tumor cells then get sufficient potential toward tumorigenesis, finishing the first step of tumor formation in the sequence. In the second step, key proto-oncogenic genes from 5q or 7 are activated due to either genetic or nongenetic reasons, such as gain-of-function mutations, gene regulations, or cytogenetic gains or the sustained microenvironment alterations around the tumor cells. Proto-oncogene mutation here is not a necessary condition. The tumor cells at this step will transit from previous latent state to present, from being local to more opt to proliferate and metastasize; the general results include a sudden increase in tumor size and further destabilized microenvironment, triggering more genetic and cytogenetic denormalization. Incidentally, deactivation of the VHL pathway can itself lead to heightened activity of VEGF and PDGF. As further genetic and cytogenetic events accumulate and more signaling pathways are deregulated, the tumor moves into the third step of progression, eventually becoming invasive and metastatic. We believe that this model describes the genetic progression of the majority of ccRCC tumors, although VHL-pathway-independent mechanism of the tumorigenesis is not excluded for a minority of tumors (Figure 5, right-hand side). Candidate tumor suppressor genes and/or oncogenes in this process were summarized in Table 3, based on the literature and our experiences. Some of these genes were well studied. Examples are the RASSF1 gene on 3p21.3 [26], FHIT on 3p14.2 [51], BHD gene on 17p11.2 [38, 39], and AKT1 gene on 14q32 [40]. The rest of genes were selected based on either literature or results of our internal gene expression profiling. 

## Supplementary Material

Supplementary Figure S1 is the summary plot when cytogenetic alteration profiles between Stage 1a and Stage 1b tumors were compared and Figure S2 is the plot when copy number alteration profiles separated in two groups by various clinical features were compared.
Supplementary Table 1 is the list of identified cytogenetic bands in which patients' cytogenetic scores were significantly associated with patients' survivals when fitting survival models and Supplementary Table 2 is the list of identified cytogenetic bands in which a formal test was significant when comparing copy number alterations between two groups of patients based on various clinical features.

## Figures and Tables

**Table 1:** Summary of clinicopathological characteristics of ccRCC samples.

	#Samples							
Studies	Current		Katte et al. 09 [14]	Beroud et al. 96 [13]	Gunawan et al. 01 [15]	Toma et al. 08 [19]	Yoshimoto et al. 07 [20]	Total
Study Size	42		246	118	118	22	26	572
*Gender*								
M	14		170	81	60	13	21	359
F	22		76	37	58	9	5	207
Total	36		246	118	118	22	26	566

*Age *(years)								
median	64.5		60.4	62	64	59	25	62
range	39–85		24–86	26–82	32–81	35–80	46–85	24–86

*Stage*								
pT1a	10	pT1	121	77	49	9	19	
pT1b + T2	12	pT2	25	14	12	7	2	
pT3a	9	pT3	97	22	34	5	2	
pT3b + T4	7	pT4	3	5	23	1	2	
Total	38		246	118	118	22	25	567

*Fuhrman Grade *								
1	3							
2	12	I	29	13	42	2	2	
2-3	5	II	106	61	63	15	9	
3	10	III	84	33	12	5	10	
3-4	8	IV	27	11	0	0	5	
4	2							
Total	40		246	118	117	22	26	569

*Size* (cm)								
median	5		6.6	6.6	6.67	NA	NA	6.6
range	1.1–12.5		1–19	1–16	1.5–25	NA	NA	1–25

NA: data not available.

**Table 2:** Selected large-scale cytogenetic studies of ccRCC tumors.

Studies		Klatte et al. 09	Beroud et al. 96	Gunawan et al. 01	Toma et al. 08	Yoshimoto et al. 07	Current	Total
Methods		GPG	qPCR	G-Banding	SNP10K	BACPAC	SNP100K	
# samples		246	118 (cc only)	118	22	26	42	572
Event	Stage	Number of events/Total Number of samples = percent						

−3p	S1	77/121 = 64%	52/77 = 68%	48/49 = 98%			19/22 = 86%	196/269 = 73%
	S2	17/25 = 68%	10/14 = 71%	12/12 = 100%			2/2 = 100%	41/53 = 77%
	S3	53/97 = 55%	15/22 = 68%	33/34 = 97%			14/15 = 93%	115/168 = 68%
	S4	0/3 = 0%	2/5 = 40%	23/23 = 100%			1/1 = 100%	26/32 = 81%
	Sum	147/246 = 60%	79/118 = 67%	116/118 = 98%	20/22 = 91%	21/26 = 81%	36/40 = 90%	419/570 = 74%

+5q	S1			28/49 = 57%			10/22 = 45%	38/71 = 54%
	S2			6/12 = 50%			2/2 = 100%	8/14 = 57%
	S3			23/34 = 67%			7/15 = 47%	30/49 = 61%
	S4			10/23 = 43%			0/1 = 0%	10/24 = 42%
	Sum	82/246 = 33%		67/118 = 57%	10/22 = 45%	15/26 = 58%	19/40 = 47%	193/452 = 43%

−14q	S1	24/121 = 20%	19/77 = 25%	29/49 = 59%			5/22 = 23%	77/269 = 29%
	S2	8/25 = 32%	4/14 = 29%	9/12 = 75%			0/2 = 0%	21/53 = 40%
	S3	36/97 = 37%	8/22 = 37%	22/34 = 65%			7/15 = 47%	73/168 = 43%
	S4	0/3 = 0%	3/5 = 60%	14/23 = 61%			0/1 = 0%	17/32 = 53%
	Sum	68/246 = 28%	34/118 = 29%	74/118 = 63%	8/22 = 36%	9/26 = 35%	12/40 = 30%	205/570 = 36%

+7		64/246 = 26%		22/118 = 19%	7/22 = 32%	9/26 = 35%	17/40 = 42%	119/452 = 26%
−8p		49/246 = 20%		39/118 = 34%			10/40 = 25%	98/404 = 24%
−6q		42/246 = 17%		28/118 = 24%	6/22 = 27%	8/26 = 31%	7/40 = 17%	91/452 = 20%
−9p		40/246 = 19%		28/118 = 24%	7/22 = 32%	5/26 = 19%	7/40 = 18%	87/452 = 19%
−4p		32/246 = 15%		17/118 = 14%			2/40 = 5%	51/404 = 13%

**Table 3:** Summary of suspected or validated genes for ccRCC on selected cytobands.

Event	1st occurred in Stage	Unique to CC	Proposed functions	Candidate Genes	References
−3p	Early	Yes	Initiation	VHL(3p25), RASSF1(3p21.3), FHIT(3p14.2), ROBO1(DUTT1, 3p12)	[4, 26–29]

+5q	Early	Yes	Differentiation & Promotion Transition from S1a to S1b	LOX(5q23), TGFBI(5q31),	
				PTTG1(5q35.1), DUSP1(5q34),	[30]
				DOCK2(5q35.1),	[16]
				PDGFRB(5q31-32)	

−14q	Early	No	Initiation, Metastasis, progression, malignant transition	HIF1A(14q21–q24), AKT1(14q32.32),	
				EGLN3(PHD3, 14q13.1),	[31]
				IGHV3(14q32.33),	
				SERPINA5(14q32)	

+7	Early	No	Proliferation Vascular Invasion	HGF(7q21.1), MET(7q31),	
				EPO(7q22), VGF(7q22), EGFR(7p12),	[32]
				IGFBP1(7p13-p12),	
				HIG2(7q32), PDGFA(7p22)	

−8p	Modest Early	Yes	Initiation?	FGF17(8p21), ANGPT2(8p23),	[33, 34] [35–37]
				DEFB1(8p23),	
				EGR3(8p23–p21)	

All others	Later	Varied		MYC(8q24.21), BHD (17p11.2),	[38–42]
				S100A4(1q21), RASSF5(1q32.1),	
			Growth Necrosis	FGFBP1(4p16-p15), CDKN2A(9p21),	
			Angiogenesis	FGFBP2(4p16), VEGFA(6p12),	
			Invasion	VEGFC(4q34.1–3), CP(3q23–q25),	
			Other unknown	ENPP2(8q24.1), AURKA(20q13.2-3),	
				NDUFA4L2(12q13), CCND1(11q13),	
				CA9 (9p13-p12)	
