# Supplementary material for: A Comprehensive Study of Progressive Cytogenetic Alterations in Clear Cell Renal Cell Carcinoma and a New Model for ccRCC Tumorigenesis and Progression
Source: Adv Bioinformatics. 2010 Jul 5;2010:428325. doi: 10.1155/2010/428325 (PMC2909727; doi:10.1155/2010/428325)
Supplement: Supplementary file 1 — Supplementary Figure S1 is the summary plot when cytogenetic alteration profiles between Stage 1a and Stage 1b tumors were compared and Figure S2 is the plot when copy number alteration profiles separated in two groups by various clinical features were compared. Supplementary Table 1 is the list of identified cytogenetic bands in which patients' cytogenetic scores were significantly associated with patients' survivals when fitting survival models and Supplementary Table 2 is the list of identified cytogenetic bands in which a formal test was significant when comparing copy number alterations between two groups of patients based on various clinical features. [file 428325.f1.pdf]

Supplementary Materials

Supplementary Figure S1. Compare profiles of cytogenetic alterations between Stage 1a and Stage 1b tumors. Cytogenetic alteration profiles for the 10 Stage 1a tumors (A), 12 stage 1b tumors (B) and the Student's t statistics between the summarized cytogenetic gains or losses for each cytoband (C). A statistic larger than 2 or less than -2 is regarded to be significant, with non grey colors. Except that there are more losses on 3p, 14q and more gains on 5q, the profiles at the rest regions are similar. (D-F) Cytogenetic alteration profiles of tumor groups with small (<3cm), medium and large (>7cm) sized tumors. Apart from the similar profiles for all three groups, +5q does not occur in small tumors, .

Supplementary Figure S2. A) Plot of averaged p-values of 100 survival models fitted on the cytogenetic alteration scores. To increase the prediction robustness, 100 models on random selected subset of patients were used for averaging the transformed p-values. Chromosome 13 losses are among the events that are most associated with patients' survival. Other associated regions include 1q, 4p and 6q, as summarized in Table 2, using a cutoff value of 0.05 on the model p-values.

B-J) Plot of p-values of studentized t-test for differences of means of cytogenetic alteration scores between patient groups based on selected clinical features. Comparisons were made on (B) VHL mutation status (yes-no), (C) gender (male or female), (D) Tumor grade (high-low), tumor stage (early-late), tumor size (small-large), sarcomatoid elements (None-Partial/Complete), gross tumor necrosis

(yes-no), renal vein invasion (yes-no), I) vascular invasion (yes-no). Cutoff value was set to be 0.05. The p-values were calculated by two sided t.test. Cytobands with test p-values larger than 0.05 are colored in grey.
