# Supplementary material for: A Comprehensive Study of Progressive Cytogenetic Alterations in Clear Cell Renal Cell Carcinoma and a New Model for ccRCC Tumorigenesis and Progression
Source: Adv Bioinformatics. 2010 Jul 5;2010:428325. doi: 10.1155/2010/428325 (PMC2909727; doi:10.1155/2010/428325)
Supplement: Supplementary file 2 [file 428325.f2.pdf]

Supplementary Table1: Identified cytogenetic bands with which patients' cytogenetic scores are significant associated with patients' survivals when fitting survival models.

chr	Cyto	Coeff[1,4]	p[2]	Signif[3]	chr	cyto	Coeff[1,4]	p[2]	Signif[3]
1	1p36	-0.157	0.0311	*	7	7p21	0.093	0.0423	*
	1p35	-0.475	0.00655	**		7q21	0.0911	0.0391	*
	1q21	0.21	0.0221	*		7q31	0.102	0.0353	*
	1q23	0.129	0.02	*		7q32	0.223	0.0408	*
	1q25	0.121	0.0147	*		7q35	0.195	0.0263	*
2	2q23	-1.58	0.0483	*	8	8q11	0.226	0.0486	*
	2q24	-0.366	0.0444	*		8q12	0.197	0.0313	*
4	4p15	-0.207	0.00292	**		8q21	0.137	0.0447	*
	4p14	-0.771	0.00092	***	13	13q12	-0.161	0.00251	**
	4p13	-0.425	0.00635	**		13q13	-0.171	0.00214	**
	4q12	-0.247	0.0449	*		13q14	-0.135	0.00188	**
	4q13	-0.179	0.025	*		13q21	-0.177	0.00054	***
	4q21	-0.247	0.00519	**		13q22	-0.245	0.000886	***
6	6q12	-0.25	0.0195	*		13q31	-0.188	0.000937	***
	6q14	-0.13	0.0303	*		13q32	-0.274	0.00092	***
	6q21	-0.157	0.0077	**		13q33	-0.173	0.000833	***
	6q22	-0.119	0.00217	**					

[1.] Averaged over coefficients of 100 models. [2]Averaged over p-values of 100 models.
[3] Significance codes: 0 '***' 0.001 '**' 0.01 '*' 0.05 '.' 0.1 ' ' 1.
[4] The absolute value means that for each additional copy number score increase, the survival probability increases (when it is positive) or decreases (when it is negative).

Supplementary Table 2: Identified cytogenetic bands with which a formal test is significant when comparing copy number alterations between two groups of patients based on clinical features.

Factors	Compare	n	cytobands
**VHL Mutation**	No<br>Yes	17<br>23	None
**Gender**	F<br>M	22<br>14	1q32-43, 4q12-3, 5q31, 7p22, 7p15-q36, 8p23-1, 9p13, 9q31
**Tumor Grade**	Low (1-2)<br>High (3-4)	15<br>20	1p36, 2p25-14, 2p12-q12, 2q14, 3p11-q13, 4q21-2, 4q24, 4q26-7, 4q31-3, 6q16-21, 7q32, 7q34-6, 8q13, 8q23, 9p24-3, 9p21, 9q31, 9q33, 12p13-q24, 17q22-4, 18q12-23, 19p12-q13, 20p12, 20q11-3
**Tumor Stage**	Early (1-2)<br>Late (3-4)	24<br>16	1p12-q43, 2p25-15, 2p12, 2q11, 3q27, 4q22, 4q26, 8q11-24, 9p24-13, 9q21-31, 9q33, 10p15, 12p13-1, 12q15, 12q23-4, 16q13, 17q22-4, 18q22, 19q12, 20q11-3
**Tumor Size (4cm)**	Small<br>large	14<br>25	1q21-32, 1q42-3, 2p25, 4q21-34, 5p15, 5q12, 5q21-35, 6q13, 9p24, 9p21-13, 9q21, 9q31, 9q33, 10q23, 12p13, 12p11, 12q23-4, 14q11-2, 17q24, 19p13-q13, 20q11, 20q13, 21q11
**Sarcomatoid Elements**	No<br>Yes	15<br>6	1q21, 1q23-31, 1q41, 2q31-3, 3p26, 3p24-14, 12p12-q12, 12q14-5, 13q34, 14q11, 16q13, 16q24
**Gross Tumor Necrosis**	No<br>Yes	11<br>8	3p23, 3q21-8, 5q22-35, 7q22-32, 7q34-6, 8q12, 8q21, 8q23-4, 11p14, 11p12, 21q11-22
**Renal Vein Invasion**	NO<br>Yes	22<br>13	1p12-q21, 1q23-42, 2p12, 2q12-3, 3q21-2, 3q28, 4q22, 6p11, 8q13, 9p23-13, 9q31, 9q33, 10p15, 12p13-1, 12q14-5, 12q22-3, 13q34, 14q11, 14q22-32, 16q13, 17q24, 18q11-23, 20q11-3
**Vascular Invasion**	No<br>Yes	17<br>13	1p12-q21, 2q12-3, 3q21-3, 3q27-8, 7p22, 7q22, 7q36, 8q13, 9p24-1, 9q33, 10p15-4, 10p12-1, 12p13-q24, 14q12, 14q23-31, 15q24, 16q13, 17q23-5, 18q12-23, 19p13-q13, 20p13, 20q11-3
